# Carbonic Anhydrases: Different Active Sites, Same Metal Selectivity Rules

**DOI:** 10.3390/molecules29091995

**Published:** 2024-04-26

**Authors:** Nikoleta Kircheva, Silvia Angelova, Todor Dudev

**Affiliations:** 1 Institute of Optical Materials and Technologies “Acad. J. Malinowski”, Bulgarian Academy of Sciences, 1113 Sofia, Bulgaria; nkircheva@iomt.bas.bg (N.K.); sea@iomt.bas.bg (S.A.); 2 University of Chemical Technology and Metallurgy, 8 St. Kliment Ohridski Blvd, 1756 Sofia, Bulgaria; 3 Faculty of Chemistry and Pharmacy, Sofia University “St. Kliment Ohridski”, 1164 Sofia, Bulgaria

**Keywords:** carbonic anhydrase, metalloenzyme, metal competition, DFT calculation

## Abstract

Carbonic anhydrases are mononuclear metalloenzymes catalyzing the reversible hydration of carbon dioxide in organisms belonging to all three domains of life. Although the mechanism of the catalytic reaction is similar, different families of carbonic anhydrases do not have a common ancestor nor do they exhibit significant resemblance in the amino acid sequence or the structure and composition of the metal-binding sites. Little is known about the physical principles determining the metal affinity and selectivity of the catalytic centers, and how well the native metal is protected from being dislodged by other metal species from the local environment. Here, we endeavor to shed light on these issues by studying (via a combination of density functional theory calculations and polarizable continuum model computations) the thermodynamic outcome of the competition between the native metal cation and its noncognate competitor in various metal-binding sites. Typical representatives of the competing cations from the cellular environments of the respective classes of carbonic anhydrases are considered. The calculations reveal how the Gibbs energy of the metal competition changes when varying the metal type, structure, composition, and solvent exposure of the active center. Physical principles governing metal competition in different carbonic anhydrase metal-binding sites are delineated.

## 1. Introduction

Carbonic anhydrases (CAs) constitute a large family of metalloenzymes which catalyze the reversible conversion of carbon dioxide and water to bicarbonate and hydrogen ions:

CO_2_ + H_2_O ↔ HCO_3_^−^ + H^+^

**Figure 1:**
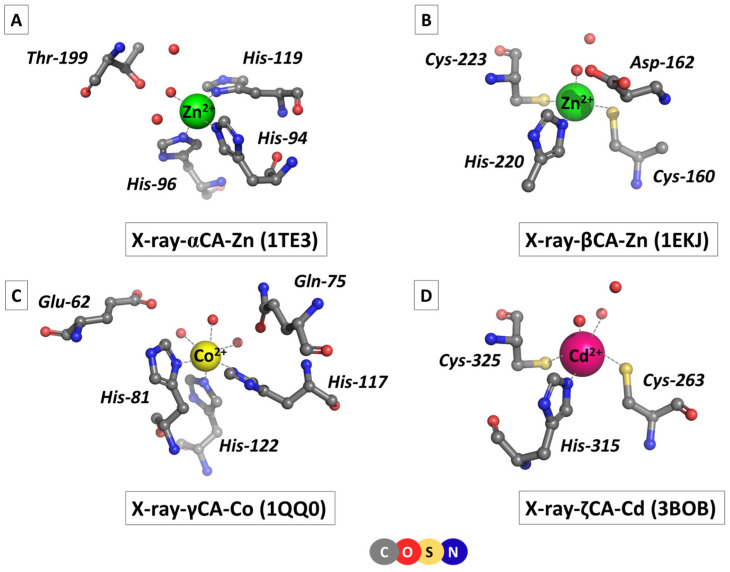
Structures of metal-binding sites of: (**A**) α-, (**B**) β-, (**C**) γ-, and (**D**) ζ-carbonic anhydrases as taken from X-ray crystallographic studies given in parentheses.

CAs are ubiquitous for the organisms belonging to all three domains of life, namely, *Archaea*, *Bacteria*, and *Eukarya*, where the enzyme plays a crucial role in a number of essential vital processes, such as maintaining acid–base homeostasis, regulating fluid balance and cellular pH, participating in CO_2_ transport, carbon fixation, respiration and photosynthesis, and influencing the hemoglobin function and cell metabolism, to name but a few [1,2,3,4,5,6,7]. Several families of carbonic anhydrases have been identified, among which αCA (found in humans and other mammals), βCA (mostly in plants), γCA (*Bacteria* and *Archaea* domains), and ζCA (Diatoms/phytoplankton) are the best functionally and structurally characterized [1,5,6,7,8]. All CAs contain a mononuclear metal-binding site which plays an indispensable role in enzyme performance: a polarized/ionized metal-bound water molecule acts as a nucleophile attacking the incoming CO_2_ [9]. It should be noted that although the mechanism of the catalytic reaction is similar, the CA families have no common ancestor and do not exhibit significant resemblance in the amino acid sequence or overall 3D structure, thus being considered as a paradigm of convergent evolution. Metal-binding sites of different classes of CAs are not identical either: they vary in the type of the metal cofactor and the arrangement and composition of its ligation sphere. This is demonstrated in Figure 1, which depicts the structure of metal-binding sites of some typical representatives of α-, β-, γ-, and ζCA as derived from X-ray crystallographic studies, where the corresponding PDB Entry is given in parentheses.

αCA comprises a Zn-binding site where the Zn^2+^ cation is tetrahedrally coordinated to three histidine side-chain ligands and a catalytic (activated) water molecule (Figure 1A; PDB code 1TE3 [10]; *Homo sapiens*). A second-shell threonine amino acid residue and two water molecules complement the architecture of the active center. βCA is also a zinc metalloenzyme but the composition of the metal-binding site differs from that of its αCA counterpart: a histidine residue and two cysteine side chains, in addition to a water ligand, orbit the metal cation in a distorted tetrahedral arrangement (Figure 1B; PDB entry 1EKJ [11]; common pea, *Pisum sativum*). The catalytic water molecule is hydrogen-bonded to another water ligand and an aspartate residue from the metal’s second coordination layer. Another type of metal-binding site has been observed in γCA (PDB code 1QQ0 [12]; methanogenic archaeon, *Methanosarcina thermophila*). First of all, the metal cofactor is not Zn^2+^ but another transition metal—Co^2+^ or Fe^2+^ [6,12]. Recent investigations have revealed that the biologically relevant metal cofactor is Fe^2+^, although the enzyme also exhibits significant activity with Co^2+^ [6]. Not surprisingly, the first-shell ligation sphere, formed by three histidine and three water ligands, is arranged in an octahedral fashion (Figure 1C) in accordance with the stereochemical requirements of the metal cation (Co^2+^ in the 1QQ0 structure; Figure 1C). Side chains of a glutamate and glutamine from the metal’s second coordination shell are hydrogen-bonded to water molecules from the primary coordination layer. Much to the surprise of the scientific community, the active site of ζCA in some marine diatoms has been found to contain Cd^2+^ as a cofactor [7,8]. Direct metal ligands—a histidine, two cysteines, and two waters—coordinate the Cd^2+^ cation in a semi-square pyramidal fashion (Figure 1D; PDB code 3BOB [8]; marine diatom, *Thalassiosira weissflogii*). A second-shell water forms hydrogen bonds with its first-shell counterparts.

Notably, although different in structure and composition, the metal-binding sites in diverse CA families have evolved to fulfil the same task—the reversible hydration of CO_2_—quite efficiently. In the course of the 3–4 billion years of organism evolution, the physicochemical properties of the metal cations and their bioavailability in the environment have played a pivotal role in choosing the proper metal cofactor to accomplish the assignment. Intriguing questions arise: How do different CAs select their cognate metal cofactor from the surrounding fluids teeming with other metal cations? How well is the native metal in the active site protected from being replaced by other metal species from the local environment? What kind of selectivity rules apply for these various metal-binding sites?

Here, we endeavor to address these questions by exploring the thermodynamic outcome of the competition between the native metal cation and its noncognate competitor in various CA model metal-binding sites. Typical representatives of competing cations from the cellular environments of the respective classes of carbonic anhydrases were considered (see the “Results” section below). Density functional theory (DFT) calculations in combination with polarizable continuum model (PCM) computations were employed (see the “Methods” section). The competition between the cognate metal and its rival can be expressed in terms of the Gibbs energy, ΔG^ε^, for replacing the native metal cation bound to the protein by its competitor:

[Comp^2+^-aq] + [Nat^2+^-protein] → [Comp^2+^-protein] + [Nat^2+^-aq] (1)

In Equation (1), [Comp^2+^/Nat^2+^-protein] and [Comp^2+^/Nat^2+^-aq] represent the metal ion (competitor or native) bound to protein ligands inside the metal-binding cavity and unbound in aqueous solution, respectively. Buried and solvent-accessible metal-active centers are characterized by an effective dielectric constant, ε, of ~4 and ~30, respectively, whereas bulk aqueous solvent is represented by ε = 78. A positive ΔG^ε^ implies a Nat^2+^-selective site, whereas a negative value implies a Comp^2+^-selective one. To delineate the key factors underlying the Comp^2+^ versus Nat^2+^ competition, we assessed how the Gibbs energy for Equation (1) changed when varying the metal type, structure, composition, and solvent exposure of the metal binding site. Physical principles governing the metal competition in different CAs active sites are delineated. It should be noted that the aim of the present calculations is to yield reliable trends in the Gibbs energy changes when varying structural parameters of the metal binding centers rather than to reproduce the absolute Gibbs energies of the metal competition. Such an approach has proven reliable in the study of other biological systems, such as enzymes [13,14], signaling proteins [15], and ion channels [16].

## 2. Results

### 2.1. αCA

**Figure 2:**
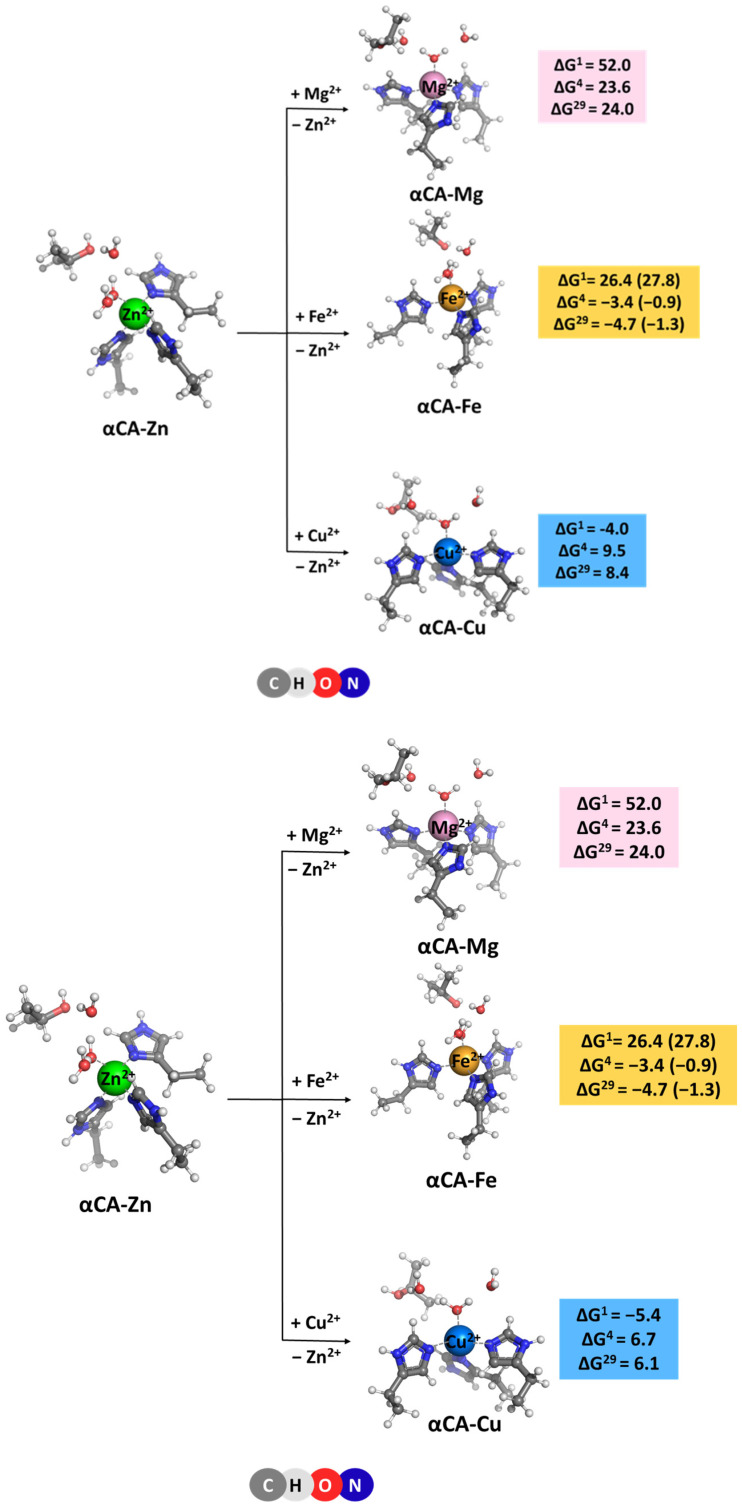
M062X/6-311++G(d,p) fully optimized structures of α-carbonic anhydrase—metal complexes and Gibbs energies (in kcal/mol) of metal exchange in different dielectric media. Superscripts 1, 4, and 29 denote gas phase, buried metal-binding site, and solvent-accessible metal center, respectively. Numbers in parentheses refer to rigid metal-binding sites.

The competition between the native Zn^2+^ cation and other biologically relevant metal cations present in cellular fluids of mammals, such as Mg^2+^, Fe^2+^, and Cu^2+^ [17] was studied. The structures of the fully optimized metal complexes are shown in Figure 2. The optimized structure of the model Zn^2+^-binding site follows closely that of the initial X-ray construct taken from the pdb file (entry 1TE3 [10]; Figure 1A). As seen, the overall shape of the binding site, where the metal is tetrahedrally coordinated to four first-shell ligands, is preserved during the optimization process. The optimization of “rival” metal complexes does not alter the general structure of the binding site either, since in all cases, the tetrahedral arrangement of the immediate metal’s surrounding is retained (Figure 2). The metal–ligand bond lengths, however, differ (though not very drastically) as their mean value decreases from 2.028 Å in the Zn^2+^ complex to 2.009 Å in its Cu^2+^ counterpart, but increases to 2.040 and 2.075 Å in the Mg^2+^ and Fe^2+^ constructs, respectively.

The Gibbs energies of metal exchange reaction (2) in different dielectric environments are also given in Figure 2. Notably, evaluating the Gibbs energies in different types of dielectric media (gas phase, ΔG^1^, vs. condensed media, ΔG^4/29^) allows for the separate accounting of different effects influencing the above equilibrium. Thus, in the gas phase, electronic effects govern the reaction outcome. Zinc cation, being a stronger Lewis acid than Mg^2+^ and Fe^2+^, forms more stable complexes and outcompetes these adversaries in the gas phase evidenced by positive ΔG^1^s. It should be noted that the active site of αCA comprising three nitrogen-containing ligands (i.e., histidines) is especially unfavorable for the Mg^2+^ binding since this “hard” metal cation has stronger preference to “hard” oxygen-containing ligands (e.g., aspartates/glutamates or backbone carbonyls) rather than to “softer” nitrogen- or sulfur-containing ligands. Copper cation, however, is more competitive than Zn^2+^ and yields negative ΔG^1^ of the Zn^2+^ → Cu^2+^ exchange. On the other side, however, the solvation effects, which are quite strong, especially for doubly charged metals and metal complexes, may substantially affect the reaction course. These can either attenuate the gas-phase Gibbs energy, ΔG^1^, which for the αCA-Mg construct decreases from 52.0 kcal/mol to ΔG^4/29^ = 23.6/24.0 kcal/mol, or reverse the reaction direction for the other two metals. Thus, Fe^2+^ appears more competitive than Zn^2+^ in a protein environment (ΔG^1^ of 26.4 kcal/mol deceases to ΔG^4/29^ of −3.4/−4.7 kcal/mol) whereas Cu^2+^ loses out against the “native” Zn^2+^ in condensed media (ΔG^1^ of −4.0 kcal/mol increases to ΔG^4/29^ of 9.5/8.4 kcal/mol). These effects can be mainly attributed to the substantial differences in the Gibbs energies of solvation of the metal cations (see “Methods” section) involved in Reaction (2). The Gibbs energy gain upon Zn^2+^ release to the bulk (ΔG_solv_ = −484.6 kcal/mol; right-hand side of Equation (2)) exceeds the desolvation penalty for the competing species (ΔG_solv_ = −455.5 and −456.4 kcal/mol for Mg^2+^ and Fe^2+^, respectively; left-hand side of Equation (2)), thus favoring the straightforward reaction for the respective Zn^2+^ → Mg^2+^ and Zn^2+^ → Fe^2+^ substitutions. Conversely, since the desolvation penalty for Cu^2+^ ion (−498.7 kcal/mol) surpasses the Gibbs energy benefit upon Zn^2+^ liberation, the Zn^2+^ → Cu^2+^ exchange appears unfavorable in a protein environment (positive ΔG^4/29^).

[M^2+^-aq] + [αCA-Zn^2+^] → [αCA-M^2+^] + [Zn^2+^-aq], M = Mg, Fe or Cu (2)

The results presented suggest that the type of protein binding site—buried (modeled with ε = 4) or solvent-accessible (modeled with ε = 29)—has little effect on the thermodynamics of the exchange process as ΔG^4^ and ΔG^29^ fluctuate in narrow limits for the respective reactions.

The effect of binding site flexibility on metal selectivity was also studied. The values presented for the Gibbs energies discussed so far were evaluated for modeled flexible binding sites where the metal ligands were allowed to relax and find their optimal position during the optimization procedure. An “experiment” was performed with a rigid/inflexible binding site, where the ligands in the non-cognate metal complex (αCA-Fe^2+^) were denied relaxation and stayed “frozen” at the initial positions of the starting αCA-Zn^2+^-derived structure during the optimization. The results are shown in parentheses in Figure 2. The calculations suggest that rigidifying the binding site of the native metal (Zn^2+^ in our case) increases its competitiveness with respect to a rival metal species (Fe^2+^ in this case), as evidenced by the increased ΔG^4/29^.

### 2.2. βCA

**Figure 3:**
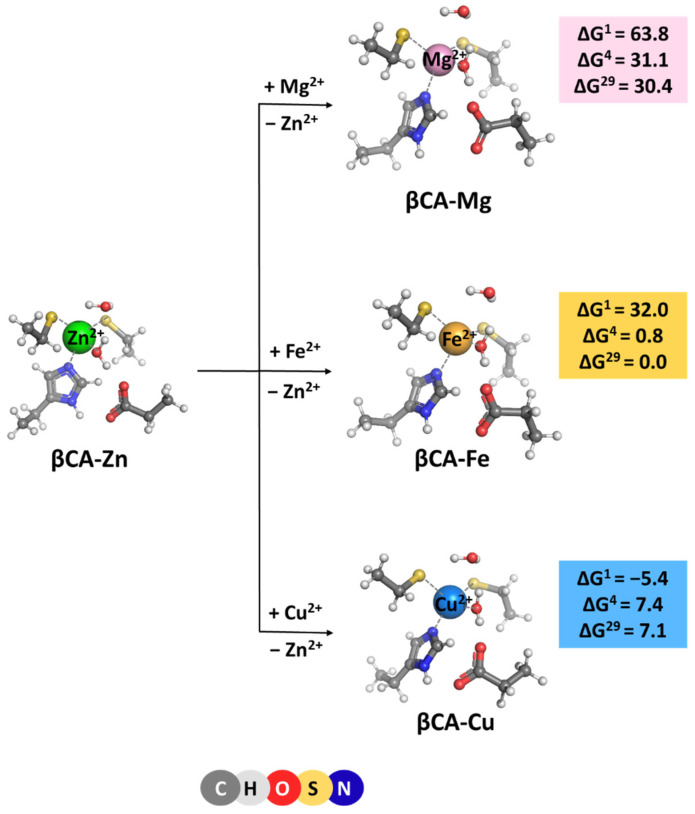
M06-2X/6-311++G(d,p) fully optimized structures of β-carbonic anhydrase—metal complexes and Gibbs energies (in kcal/mol) of metal exchange in different dielectric media. Superscripts 1, 4, and 29 denote gas phase, buried metal-binding site, and solvent-accessible metal center, respectively.

β-Carbonic anhydrase is found predominantly in plants. Therefore, our models were derived from the X-ray structure (PDB entry 1EKJ, [11], 1.93 Å resolution) of plant βCA. The native metal cation in βCA is, again, Zn^2+^. The amino acid ligation sphere, however, differs from that in its α-counterpart: instead of three histidines in αCA, a histidine and two cysteine ligands orbit the metal cofactor in βCA (Figure 1B). In both cases, the central metal cation is (semi) tetrahedrally coordinated to the first-shell ligands. The optimized structure of the model Zn^2+^-binding site is shown in Figure 3. As seen, the overall shape of the metal center is preserved during the optimization. The Zn^2+^ major competitors in plants appear to be Mg^2+^, Fe^2+^, and Cu^2+^ which are present in appreciable quantities in the cellular environment [18,19]. Thus, complexes of these rival metals were also modeled (after substituting for the native Zn^2+^ cation; see “Methods” section) and subsequently optimized (Figure 3). The optimization of “rival” metal complexes does not alter the overall structure of the binding site as, in all cases, the (semi) tetrahedral arrangement of the immediate metal’s surrounding is retained (Figure 3). It should be noted that the metal complexes are bulkier than the respective αCA-metal constructs (see above): the mean value of the metal–ligand bond lengths for the Zn^2+^ complex is 2.228 Å, 2.214 Å for its Cu^2+^ counterpart, and 2.256 and 2.306 Å for the respective Fe^2+^ and Mg^2+^ structures.

The Gibbs energies for the metal exchange in βCA metal centers, reaction (3) were evaluated and are presented in Figure 3. Generally, the trends of changes in ΔGs of the metal exchange reactions in βCA metal-binding sites are similar to those observed for their αCA counterpart. The least competitive to the cognate Zn^2+^ cation is Mg^2+^ (highest positive Gibbs energies of metal exchange in a protein environment) followed by Cu^2+^ (lower positive ΔG^4/29^) and Fe^2+^ (lowest ΔG^4/29^). The calculations imply that the zinc-binding sites in βCA are reliably protected against “foreign” invasion. Comparing the numerical data obtained for αCA and βCA, one can notice that the metal-binding site in the latter favors the Zn^2+^/Mg^2+^ and Zn^2+^/Fe^2+^ competition to a greater extent than in the former (higher positive ΔGs of metal exchange in βCA than in αCA; Figure 2 and Figure 3). This is due to the presence of two cysteine residues in the active center of βCA in place of the respective number of histidine residues in the αCA construct, which secure stronger interactions with the native Zn^2+^ than with its rival Mg^2+^ and Fe^2+^ cations. On the other hand, Cu^2+^, with a higher affinity for sulfur-containing ligands than Zn^2+^, increases (slightly) its competitiveness in βCA, although ΔGs remain positive. Again, varying the solvent exposure of the binding pocket has little effect on the metal competition.

[M^2+^-aq] + [βCA-Zn^2+^] → [βCA-M^2+^] + [Zn^2+^-aq] (M = Mg, Fe or Cu) (3)

### 2.3. γCA

**Figure 4:**
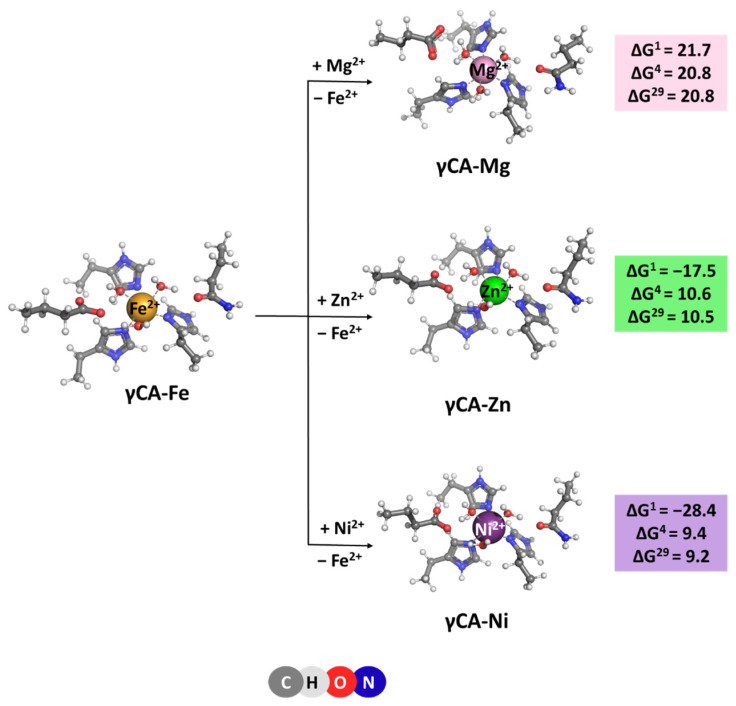
M062X/6-311++G(d,p) fully optimized structures of γ-carbonic anhydrase—metal complexes and Gibbs energies (in kcal/mol) of metal exchange in different dielectric media. Superscripts 1, 4, and 29 denote gas phase, buried metal-binding site, and solvent-accessible metal center, respectively.

The native metal for γCA is Fe^2+^ [6], which is octahedrally coordinated to six ligands—three histidine side chains and three water molecules. Optimized structures of the model metal-binding sites of the γCA-Fe complex and constructs comprising rival cations from the saline water environment such as Mg^2+^, Ni^2+^, and Zn^2+^ [20,21] are depicted in Figure 4. All complexes retain the initial octahedral coordination of the metal ion during the optimization. Gibbs energies evaluated for the competition between the cognate metal and its contestants reaction (4), are also given in Figure 4. The data presented suggest that the metal-binding site in γCA can withstand attacks from other metal species from the surrounding fluids as all Gibbs energies of metal exchange in a protein environment, ΔG^4/29^, are positive. Again, Mg^2+^ is the weakest competitor of the native metal species. Zn^2+^ and Ni^2+^ cations cannot successfully compete with Fe^2+^ either. Two major factors contribute to this result: (1) six-coordinated complexes suit Fe^2+^ better than its contestants as the latter prefer lower coordination-number constructs—usually four for Zn^2+^ and four to five for Ni^2+^ complexes; and (2) the desolvation penalty of the attacking Zn^2+^ and Ni^2+^ (484.6 and 494.2 kcal/mol, respectively; left-hand side of Equation (4)) exceeds the Gibbs energy gain of the liberated Fe^2+^ (−456.4 kcal/mol; right-hand side of Equation (4)), which favors the backward Reaction (4).

[M^2+^-aq] + [γCA-Fe^2+^] → [γCA-M^2+^] + [Fe^2+^-aq] (M = Mg, Zn or Ni) (4)

### 2.4. ζCA

**Figure 5:**
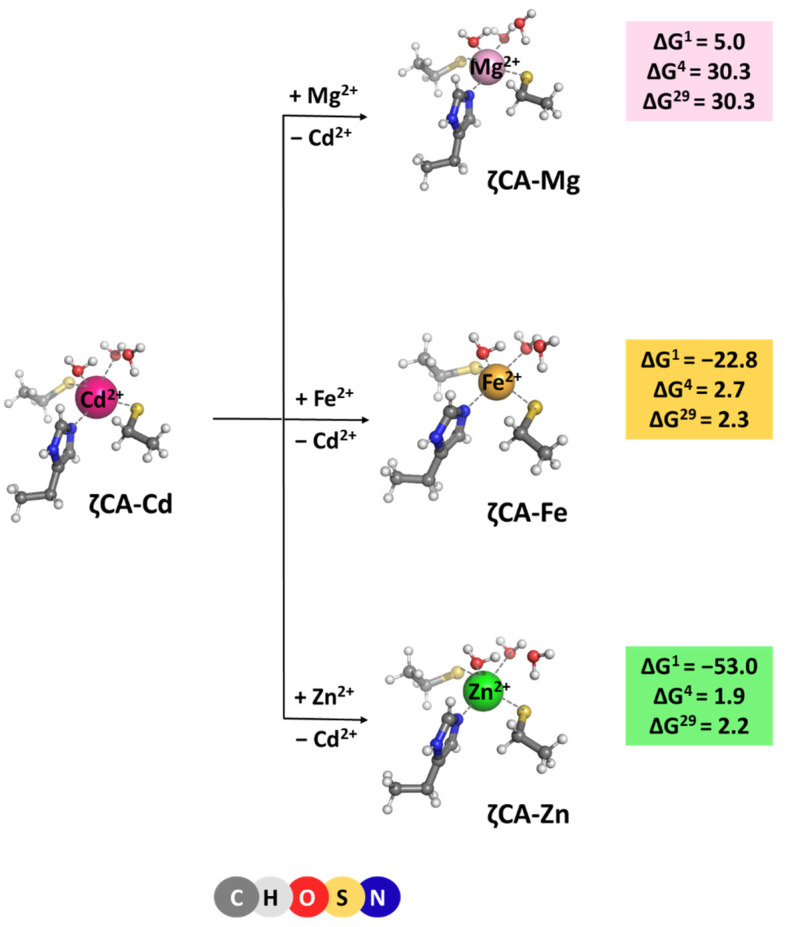
M062X/6-311++G(d,p) fully optimized structures of ζ-carbonic anhydrase—metal complexes and Gibbs energies (in kcal/mol) of metal exchange in different dielectric media. Superscripts 1, 4, and 29 denote gas phase, buried metal-binding site, and solvent-accessible metal center, respectively.

ζ-Carbonic anhydrase is a Cd^2+^ enzyme. The metal cofactor is coordinated to five ligands: a histidine and two cysteine side chains donated by the protein, and two water molecules. Optimized structures of ζCA-Cd and ζCA-Mg/Fe/Zn complexes, which all preserve the original five-coordinated ligation pattern, are presented in Figure 5 along with the Gibbs energies of the metal exchange:

[M^2+^-aq] + [ζCA-Cd^2+^] → [ζCA-M^2+^] + [Cd^2+^-aq] (M = Mg, Fe or Zn) (5)

Positive ΔG^4/29^ evaluated for all reactions indicate that Cd^2+^ is the cation of choice for the ζCA. Again, the structure of the metal-binding site (which is well adapted to the specific coordination requirements of Cd^2+^ rather than of Mg^2+^, Fe^2+^, and Zn^2+^) and the balance between the desolvation penalty for the attacking metal species and the Gibbs energy gain of the outgoing Cd^2+^ (which is in favor of the native Cd^2+^ cation) determine the outcome of the competition process.

## 3. Methods

### 3.1. Models Used

Metal-binding sites in α-, β-, γ-, and ζCA were modeled after the respective X-ray structures, deposited in the Protein Data Bank [22,23]. The metal cation and its ligands—amino acid residues and water molecules—from both the first and second coordination layer were incised from the protein structure and further modified by capping the amino acid side chains at the C^α^ atom with a methyl group. Thus, the side chains of His, Cys^−^, Asp^−^, Glu^−^, Thr, and Gln were represented by ethyl-imidazole, CH_3_CH_2_S^−^, CH_3_CH_2_COO^−^, CH_3_CH_2_CH_2_COO^−^, CH_3_CH(OH)CH_3_, and CH_3_CH_2_CH_2_CONH_2_, respectively. The coordinates of the C^α^ atoms were kept frozen during the ensuing optimization to account for the packing/rigidifying effect of the protein matrix.

### 3.2. DFT/PCM Calculations

The Gaussian 09 suite of programs [24] was employed in performing the required calculations. The most suitable combination of theoretical method/basis set was found to be the Minnesota density functional M062X method [25] in conjunction with the 6-311++G(d,p) basis set for all “light” atoms (C, H, N, O, S, Zn, Cu, Mg, Ni, and Fe), and the SDD basis set/effective core potential [26] for the “heavy” Cd. Our previous studies [14] proved it dependable in correctly reproducing the geometry of a series of representative metal structures and the Gibbs energies of metal exchange in acetate, imidazole, and glycine complexes too [27].

As a first step in the current study, the metal-loaded binding sites comprising the cognate metals (Zn^2+^ for both α- and βCA, Fe^2+^ for γCA, and Cd^2+^ for ζCA) were fully optimized. The second step was the optimization of the resulting constructs where the native metal was replaced by its rival metal species. The respective structures, as shown in Figure 2, Figure 3, Figure 4 and Figure 5, were discussed accordingly in the preceding sections. The calculations provided the electronic energies, E_el_, for each optimized metal complex. According to the performed vibrational frequency calculations (at the same M062X/6-311++G(d,p)//SDD level of theory), the located stationary points on the potential energy surface were found to be energy minima. The frequencies were scaled by an empirical factor of 0.983 [25] and employed to evaluate the thermal energies, E_th_, including zero-point energy, and entropies, S. The metal exchange Gibbs energy in the gas phase, ΔG^1^, at T = 298.15 K and 1 atm was calculated using the electronic energies and the thermodynamic properties according to the following equation:

ΔG^1^ = ΔE_el_^1^ + ΔE_th_^1^ − TΔS^1^ (6)

In Equation (6), Δ stands for the differences in E_el_, E_th_, and S between the products and reactants. The basis set superposition error for the type of metal substitution reactions modeled by Equation (1) is insignificant [28], and, therefore, it was not considered in the present calculations.

Metal-binding sites in metalloenzymes are situated in cavities of the protein structure whose dielectric properties differ from those in the bulk water [29] and exhibit characteristics closer to the low-polarity solvents [30]. Thus, condensed-phase calculations were performed in solvents emulating the dielectric properties of buried and solvent-accessible binding sites, diethyl ether (ε = 4) and propanonitrile (ε = 29), respectively. The SMD (Solvation Model based on Density) [31] version of the Polarizable Continuum Model was employed in accounting for the solvation effects by subjecting each optimized structure in the gas phase to single-point calculations in the respective solvent at the M062X/6-311++G(d,p)//SDD level of theory. The differences between the gas-phase and SMD energies were used to compute the solvation Gibbs energy, ΔG_solv_^ε^, of each metal construct. The incoming and outgoing metal species were considered to be in a bulk watery environment (ε = 78) outside the binding pocket. Hence, their experimentally determined hydration Gibbs energies [32] were used in the computations: ΔG^78^(Mg^2+^) = −455.5 kcal/mol; ΔG^78^(Zn^2+^) = −484.6 kcal/mol; ΔG^78^(Cu^2+^) = −498.7 kcal/mol; ΔG^78^(Fe^2+^) = −456.4 kcal/mol; ΔG^78^(Ni^2+^) = −494.2 kcal/mol; and ΔG^78^(Cd^2+^) = −430.5 kcal/mol. The cation exchange Gibbs energy, ΔG^ε^, in a protein binding site characterized by an effective dielectric constant ε was evaluated as:

ΔG^ε^ = ΔG^1^ + ΔG_solv_^ε^([Comp^2+^-protein]) − ΔG_solv_^ε^ [Nat^2+^-protein] − ΔG_solv_^78^([Comp^2+^-aq]) + ΔG_solv_^78^([Nat^2+^-aq]) (7)

## 4. Conclusions

The competition between the cognate metal cofactor and other metal species from the surrounding fluids in four different classes of carbonic anhydrases was studied through high-level DFT/PCM calculations. The results obtained provide a basis for delineating the major determinants of the metal selectivity in these systems. Although the structure and composition of the active sites of αCA, βCA, γCA, and ζCA are different, the metal selectivity principles, employed by the enzyme, appear to be identical for all the representatives of the carbonic anhydrase family. Thus, Mg^2+^, which is quite abundant in the cellular environment of organisms from all three domains of life, cannot compete successfully with the native metal cofactor in the active site since, unlike the cognate Zn^2+^, Fe^2+^, and Cd^2+^ cations, it has low affinity for nitrogen- and sulfur-containing protein ligands (histidines and cysteines). Also, the tetrahedral arrangement of metal-binding sites in αCA and βCA is not favorable for Mg^2+^ as it prefers octahedrally shaped ligation spheres. The symmetry of the metal-binding site also plays a substantial role in the competition between the transition metals. Thus, in γCA, the octahedrally arranged binding site strongly benefits Fe^2+^ but is not optimal for Zn^2+^ and Ni^2+^ as those cations prefer smaller number of ligands orbiting the metal cation. The same rule also applies for the Cd^2+^ center in ζCA, where its structure is appropriately adapted to its specific coordination requirements. Furthermore, the metal cation solvation is another factor which has to be taken into account in assessing the outcome of the metal competition. As shown in the preceding sections, the balance between the desolvation penalty of the attacking metal species and the Gibbs energy gain of the outgoing native metal emerges as a major determinant of the metal selectivity in the systems studied. Rigidifying the metal-binding site, thus imposing the overall geometry of the native construct on the attacking competitor, also increases the competitiveness of the cognate metal species (Figure 2; Zn^2+^/Fe^2+^ competition). On the other hand, the solvent exposure of the metal-binding site seems to be a factor of lesser importance for the selectivity process.

Notably, Fe^2+^ appears to be a strong competitor of Zn^2+^ in αCA and βCA, as evidenced by the small Gibbs energies of the metal exchange (Figure 2 and Figure 3). Thus, in some circumstances (for example, Zn^2+^ deficiency), Fe^2+^ might substitute for the native Zn^2+^ and keep the enzyme functional. In fact, Fe^2+^ is the native metal cofactor in γCA.

## Data Availability

The data presented in this study are available on request from the corresponding author.

## References

[1] Di Fiore A., D’Ambrosio K., Ayoub J., Alterio V., De Simone G. (2019). α-Carbonic Anhydrases.

[2] Henry R.P. (1996). Multiple Roles of Carbonic Anhydrase in Cellular Transport and Metabolism. Annu. Rev. Physiol..

[3] Supuran C.T. (2004). Carbonic Anhydrases: Catalytic and Inhibition Mechanisms, Distribution and Physio-Logical Roles. Carbonic Anhydrases.

[4] Occhipinti R., Boron W.F. (2019). Role of Carbonic Anhydrases and Inhibitors in Acid–Base Physiology: Insights from Mathematical Modeling. Int. J. Mol. Sci..

[5] DiMario R.J., Clayton H., Mukherjee A., Ludwig M., Moroney J.V. (2017). Plant Carbonic Anhydrases: Structures, Locations, Evolution, and Physiological Roles. Mol. Plant.

[6] Ferraroni M. (2019). γ-Carbonic Anhydrases. Carbon Anhydrases Biochemistry and Pharmacology of an Evergreen Pharmaceutical Target.

[7] Alterio V., Langella E., Viparelli F., Vullo D., Ascione G., Dathan N.A., Morel F.M.M., Supuran C.T., De Simone G., Monti S.M. (2012). Structural and Inhibition Insights into Carbonic Anhydrase CDCA1 from the Marine Diatom *Thalassiosira weissflogii*. Biochimie.

[8] Xu Y., Feng L., Jeffrey P.D., Shi Y., Morel F.M.M. (2008). Structure and Metal Exchange in the Cadmium Carbonic Anhydrase of Marine Diatoms. Nature.

[9] Lindskog S. (1997). Structure and Mechanism of Carbonic Anhydrase. Pharmacol. Ther..

[10] Fisher Z., Hernandez Prada J.A., Tu C., Duda D., Yoshioka C., An H., Govindasamy L., Silverman D.N., McKenna R. (2005). Structural and Kinetic Characterization of Active-Site Histidine as a Proton Shuttle in Catalysis by Human Carbonic Anhydrase II. Biochemistry.

[11] Kimber M.S. (2000). The Active Site Architecture of Pisum Sativum β-Carbonic Anhydrase Is a Mirror Image of That of Alpha -Carbonic Anhydrases. EMBO J..

[12] Iverson T.M., Alber B.E., Kisker C., Ferry J.G., Rees D.C. (2000). A Closer Look at the Active Site of γ-Class Carbonic Anhydrases: High-Resolution Crystallographic Studies of the Carbonic Anhydrase from *Methanosarcina thermophila*. Biochemistry.

[13] Nikolova V., Angelova S., Markova N., Dudev T. (2016). Gallium as a Therapeutic Agent: A Thermodynamic Evaluation of the Competition between Ga^3+^ and Fe^3+^ Ions in Metalloproteins. J. Phys. Chem. B.

[14] Dudev T., Cheshmedzhieva D., Doudeva L. (2018). Competition between Abiogenic Al^3+^ and Native Mg^2+^, Fe^2+^ and Zn^2+^ Ions in Protein Binding Sites: Implications for Aluminum Toxicity. J. Mol. Model..

[15] Dudev T., Grauffel C., Lim C. (2018). How Pb^2+^ Binds and Modulates Properties of Ca^2+^-Signaling Proteins. Inorg. Chem..

[16] Dudev T., Lim C. (2014). Ion Selectivity Strategies of Sodium Channel Selectivity Filters. Acc. Chem. Res..

[17] Dudev T., Lim C. (2014). Competition among Metal Ions for Protein Binding Sites: Determinants of Metal Ion Selectivity in Proteins. Chem. Rev..

[18] Schmidt S.B., Eisenhut M., Schneider A. (2020). Chloroplast Transition Metal Regulation for Efficient Photosynthesis. Trends Plant Sci..

[19] Yruela I. (2013). Transition Metals in Plant Photosynthesis. Metallomics.

[20] Bardi U. (2010). Extracting Minerals from Seawater: An Energy Analysis. Sustainability.

[21] Bazzi A.O. (2014). Heavy Metals in Seawater, Sediments and Marine Organisms in the Gulf of Chabahar, Oman Sea. J. Oceanogr. Mar. Sci..

[22] Berman H., Henrick K., Nakamura H. (2003). Announcing the Worldwide Protein Data Bank. Nat. Struct. Biol..

[23] Berman H., Henrick K., Nakamura H., Markley J.L. (2007). The Worldwide Protein Data Bank (WwPDB): Ensuring a Single, Uniform Archive of PDB Data. Nucleic Acids Res..

[24] Frisch M.J., Trucks G.W., Schlegel H.B., Scuseria G.E., Robb M.A., Cheeseman J.R., Scalmani G., Barone V., Mennucci B., Petersson G.A. (2013). Gaussian 09, Revision D. 01.

[25] Zhao Y., Truhlar D.G. (2008). The M06 Suite of Density Functionals for Main Group Thermochemistry, Thermochemical Kinetics, Noncovalent Interactions, Excited States, and Transition Elements: Two New Functionals and Systematic Testing of Four M06-Class Functionals and 12 Other Function. Theor. Chem. Acc..

[26] Andrae D., Häußermann U., Dolg M., Stoll H., Preuß H. (1990). Energy-Adjusted Ab Initio Pseudopotentials for the Second and Third Row Transition Elements. Theor. Chim. Acta.

[27] Dudev T., Nikolova V. (2016). Determinants of Fe^2+^ over M^2+^ (M = Mg, Mn, Zn) Selectivity in Non-Heme Iron Proteins. Inorg. Chem..

[28] Dudev T., Lim C. (2009). Determinants of K^+^ vs Na^+^ Selectivity in Potassium Channels. J. Am. Chem. Soc..

[29] Li L., Li C., Zhang Z., Alexov E. (2013). On the Dielectric “Constant” of Proteins: Smooth Dielectric Function for Macromolecular Modeling and Its Implementation in DelPhi. J. Chem. Theory Comput..

[30] Mertz E.L., Krishtalik L.I. (2000). Low Dielectric Response in Enzyme Active Site. Proc. Natl. Acad. Sci. USA.

[31] Marenich A.V., Cramer C.J., Truhlar D.G. (2009). Universal Solvation Model Based on Solute Electron Density and on a Continuum Model of the Solvent Defined by the Bulk Dielectric Constant and Atomic Surface Tensions. J. Phys.Chem B.

[32] Friedman H.L., Krishnan C.V., Franks F. (1973). Water: A Comprehensive Treatise.

